# Preserved memory-based orienting of attention with impaired explicit memory in healthy ageing

**DOI:** 10.1016/j.cortex.2015.10.019

**Published:** 2016-01

**Authors:** Gerardo Salvato, Eva Z. Patai, Anna C. Nobre

**Affiliations:** a Oxford Centre for Human Brain Activity, University of Oxford, Oxford, United Kingdom; b Department of Experimental Psychology, University of Oxford, Oxford, United Kingdom; c Department of Brain and Behavioural Sciences, University of Pavia, Pavia, Italy; d NeuroMi, Milan Centre for Neuroscience, Italy

**Keywords:** Long-term memory, Ageing, Contextual memory, Spatial attention, Visual perception

## Abstract

It is increasingly recognised that spatial contextual long-term memory (LTM) prepares neural activity for guiding visuo-spatial attention in a proactive manner. In the current study, we investigated whether the decline in explicit memory observed in healthy ageing would compromise this mechanism. We compared the behavioural performance of younger and older participants on learning new contextual memories, on orienting visual attention based on these learnt contextual associations, and on explicit recall of contextual memories. We found a striking dissociation between older versus younger participants in the relationship between the ability to retrieve contextual memories versus the ability to use these to guide attention to enhance performance on a target-detection task. Older participants showed significant deficits in the explicit retrieval task, but their behavioural benefits from memory-based orienting of attention were equivalent to those in young participants. Furthermore, memory-based orienting correlated significantly with explicit contextual LTM in younger adults but not in older adults. These results suggest that explicit memory deficits in ageing might not compromise initial perception and encoding of events. Importantly, the results also shed light on the mechanisms of memory-guided attention, suggesting that explicit contextual memories are not necessary.

## 1 Introduction

Neural systems supporting explicit long-term contextual memory (LTM) are often reported to decline in healthy ageing (Dennis and Cabeza, 2008, Dew and Giovanello, 2010, Fleischman et al., 2004, Old and Naveh-Benjamin, 2008). Impairments include explicit retrieval of spatial, temporal, and semantic features of contextual LTM (Chalfonte and Johnson, 1996, Kessels et al., 2007, Kessels et al., 2010, Meulenbroek et al., 2010, Old and Naveh-Benjamin, 2008). Furthermore, structural decline often affects specific brain regions, such as the frontal lobes and medial temporal structures (e.g., hippocampus), which underpin the successful encoding and retrieval of explicit LTM in younger healthy adults (Bartzokis et al., 2001, Broadbent et al., 2004, Lim et al., 1992, Persson et al., 2006, Raz, 2000, Raz et al., 2004, Squire et al., 1992, Yonelinas et al., 2001).

Evidence regarding impairment of implicit forms of contextual memory is mixed. Most studies report preserved abilities. For instance, older adults show similar advantages as younger adults for locating target objects that appear at locations compatible with their real-world placement (Neider & Kramer, 2011). They also have been shown to use contextual information to guide search in a Digit Matrix Scanning Task (Schmitter-Edgecombe & Nissley, 2002) and in contextual cueing paradigms (Howard et al., 2005, Merrill et al., 2013). However, at least one recent study using the contextual-cueing paradigm has failed to replicate benefits from implicitly learned associations (Smyth & Shanks, 2011).

It has been proposed that impairments in memory tasks might be caused by reduction in attentional or processing resources (Craik, 1986, Craik and Trehub, 1982). Decline in attention-related functions have been noted during ageing (Sommers and Huff, 2003, Trick et al., 2005, Zanto et al., 2011). However, findings on age-related modulation of attention are multifaceted (see Zanto & Gazzaley, 2014 for a recent review). Indeed, several aspects of attention such as, performing spatial orienting of attention (Gottlob and Madden, 1999, Hartley et al., 1990, Nissen and Corkin, 1985), ignoring intermodal distraction (Beaman, 2005, Bell and Buchner, 2007) and local task-switching (Verhaeghen and Cerella, 2002, Wasylyshyn et al., 2011) appear to be preserved in healthy ageing.

It is increasingly recognised that contextual and associative LTM does not merely support remembering previously learnt information. These rich representations of previous experience can also interact with visuo-spatial attention, exerting robust influence on perception and decision-making in a prospective and proactive manner (Bar, 2009, Chun and Johnson, 2011, Hutchinson and Turk-Browne, 2012, Nobre and Mesulam, 2014). In our laboratory, we have developed an experimental paradigm to investigate how contextual LTM for the location of objects in scenes can guide spatial attention to target events occurring in previously learned locations (Summerfield, Lepsien, Gitelman, Mesulam, & Nobre, 2006). We have demonstrated that scenes cueing the memory of target locations enhance excitability in visual areas in anticipation of target appearance (Stokes et al., 2012, Summerfield et al., 2011) and magnify the early visual responses to target stimuli at the remembered location (Summerfield et al., 2011). Behaviourally, this results in higher perceptual sensitivity and faster reaction times (RTs) to detect targets (Doallo et al., 2013, Patai et al., 2012, Summerfield et al., 2006). These behavioural benefits for detecting targets in remembered locations are accompanied by measures of explicit memory for target locations. Our imaging studies suggest that medial temporal lobe structures, and specifically the hippocampal system, may contribute to LTM-based orienting of attention (Stokes et al., 2012, Summerfield et al., 2006), though their causal involvement remains to be tested by interference-based methods.

Hence, the question arises of whether the functional decline of explicit contextual LTM occurring in healthy ageing and the structural decline in associated brain regions may adversely influence cognition from much earlier perceptual stages than hitherto considered. If explicit spatial contextual memories are vital in sharpening perception, is it possible that even the early stages of perception are already impaired in older individuals?

In the current investigation we tested for decline in explicit contextual LTM in healthy older participants compared to younger participants, and we investigated whether this memory decline would also compromise the ability to optimise perceptual processing according to previous experience. A close association between decline in explicit contextual memories and in the perceptual benefits based on previous experience would further implicate memory systems that support explicit contextual memory to drive proactive memory-based attention.

## 2 Materials and methods

### 2.1 Participants

The experimental protocol had ethical approval from the University of Oxford Central University Research Ethics Committee. Twenty-two right-handed healthy older adults (9 males, 13 females; ages range 62–80, *M* = 66.5, *SD* = 4.7; years of education *M* *=* 1 = 6, *SD* = 2.7) and 22 right-handed healthy younger adults (9 males, 13 females; ages range 20–38, *M* = 26.8, *SD* = 5.4; education *M* = 16.27, *SD* = 2.2) participated. They were matched on gender [χ^2^(1, *N* = 44) = 1.00; *p* = .620] and education [*t*(42) = .30; *p* = .765]. All were native English speakers, and had normal or corrected-to-normal vision, and had no previous history of mental or neurological illness. The Montreal Cognitive Assessment (MoCA) (Nasreddine et al., 2005) was administered to older participants, in order to rule out any cognitive deficits (median score 28; range 27–30). Informed consent was obtained prior to participation in the experiment, and participants were remunerated £30 for their time.

## 3 Materials & methods

### 3.1 Stimuli

The task we used in this study was a modified version of the experimental design previously developed in our laboratory (Summerfield et al., 2006). Digital photographs of complex scenes and of everyday objects were used to construct the visual stimuli. Colour photographs (1000 × 750 pixels) of indoor and outdoor scenes were obtained from the Flickr Creative Commons (Judd, Ehinger, Durand, & Torralba, 2009). The objects were selected from the SUN dataset (Xiao, Hays, Ehinger, Oliva, & Torralba, 2010). Ninety-six scenes and objects were used for the main experiment, and an additional twelve were used for familiarisation and practise trials. The object images were sized to fit within a 100 × 100 pixels transparent box (3.4° × 4.5°) when superimposed on the scene and 150 × 150 pixels transparent box (5.2° × 6.7°) when presented against a grey background. Each object was randomly assigned to one scene to create novel spatial/contextual object-scene associations. For counterbalancing purposes, two versions of object-scene combinations were prepared for each scene, with the target placed either on the left or on the right side. Across all scenes, objects were placed equiprobably in all four quadrants (upper left, lower left, upper right, lower right). Objects were not necessarily semantically related to the scene, and object placement was not always realistic.

The experiment included three different phases: learning, orienting, and explicit retrieval. An eye-tracking camera (EyeLink 1000, SR Research) was used to monitor eye movements during all phases.

### 3.2 Task and procedure

#### 3.2.1 Learning

**Fig. 1:**
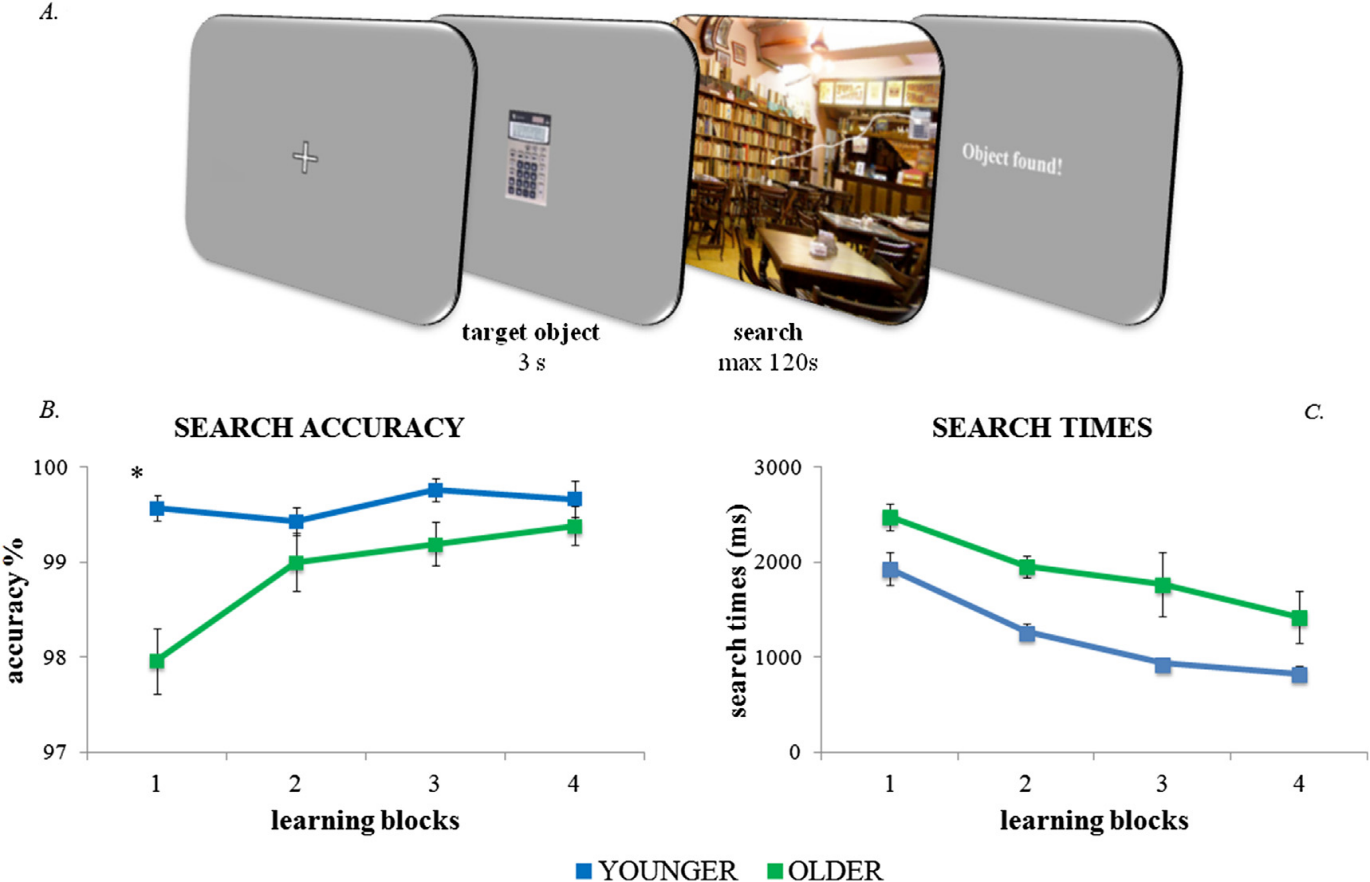
Learning: experimental design and results. (A) Schematic illustration of the task structure. An everyday object was presented for 3 sec. Then a scene containing that object appeared. Participants had 2 min to find the target. (B) As learning blocks progressed, older participants found more target objects while younger were at ceiling effect (younger in blue, older in green). (C) Search Times showed the same linear decrease over the learning session for younger and older participants, despite older participants being slower overall.

During the learning session, participants studied each scene to locate and memorise the ‘target’ object associated with it (Fig. 1A). Ninety-six scenes and their paired objects were randomly intermixed and studied over each of the four blocks. Participants were told that a given object would always appear at the same position within a given scene over the learning blocks. At the beginning of each trial, the search target was presented centrally against a grey background for 3 s. A scene containing that object then appeared on the screen. Participants overtly explored the scene to locate the target. Once they located the object, participants clicked the left mouse button to make a central white-square cursor appear. They then moved this cursor over the object, and clicked again. The search period timed out after a maximum of 120 s. If the object was correctly located (maximum cursor error: 50 pixels), participants were given positive feedback (“Object found”). If they were incorrect, or were unable to locate the object, they received negative feedback (“Object not found”). There was a variable fixation period (1000–1500 msec) between trials. Short breaks between learning blocks were also provided. As measures of forming contextual memories, we took into account the percentage of correct responses (Search Accuracy) and the mean Search Times in the four learning blocks. The Search Times were calculated as time from the scene onset and the time that the participants made their first mouse click.

Once the Learning session was completed, participants had a 30-min break. During this period they relaxed in a different room. They were engaged in a conversation with the experimenter to distract them. In order to avoid interference with the memory trace consolidation, the use of any devices or printed materials containing scenes and objects (e.g., newspapers, magazines, personal computers, tablets) was avoided.

#### 3.2.2 Orienting

**Fig. 2:**
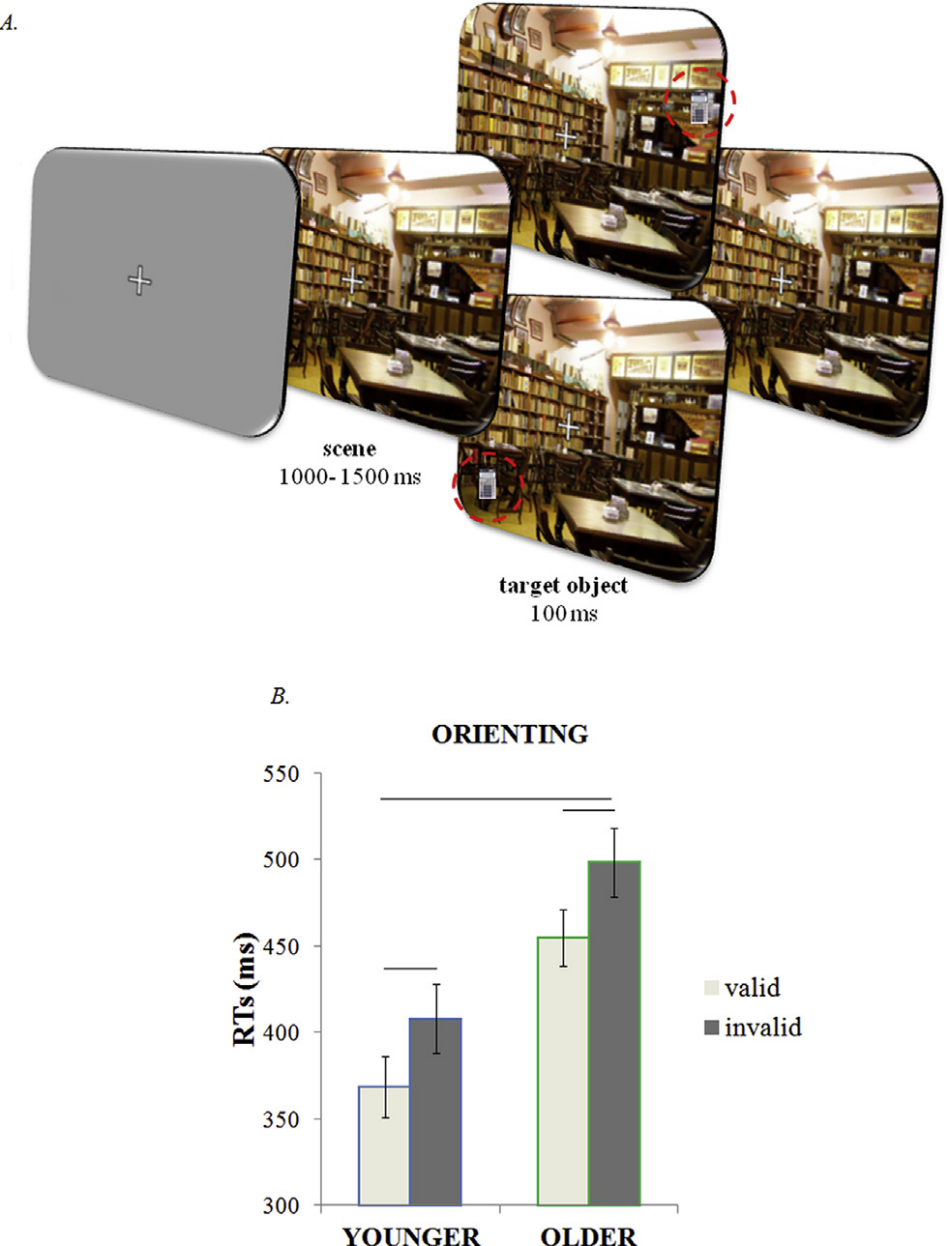
Orienting session: experimental design and results. (A) Schematic illustration of the orienting task structure. At the beginning of each trial, a cue scene previously associated with a target object appeared on the screen. After 1000–1500 msec, the target object flashed up on the scene in either a “valid” learned (here indicated in a red circle at top row) on half the trials, or in an “invalid” unlearned location (here indicated in a red circle at bottom row). Participants responded as soon as they saw the target object on the scene, but refrained from responding if a foil appeared (not shown). (B) Mean RTs for valid and invalid trials showed that older and younger participants benefited from the long-term memories to facilitate perception.

After the rest interval, participants performed a memory-guided spatial orienting task (Fig. 2A). Seventy-two of the 96 scenes were presented in randomised order. Participants viewed each of these studied scenes, and were required to make a speeded detection response when an object flashed briefly on the scene. If a stop sign appeared, they had to refrain from responding. Participants were instructed to fixate centrally throughout the task. They performed a brief practice session (12 trials) using a different set of scenes before the task to ensure they understood the instructions and could refrain from making eye movements. At the beginning of each trial, a fixation cross (1000–1500 msec) indicated that a scene was about to appear. One of the studied scenes then appeared, without any target object present. After a variable interval (1000–1500 msec), an object was briefly (100 msec) superimposed on the scene. In 64 of the learned scenes, this object was the target object associated with the same scene from the learning phase; in the remaining 8 scenes, a foil (a yellow hexagonal stop sign with black lettering) appeared instead of the target object. Participants were required to respond to the target objects with a left button mouse click as quickly as possible, but to refrain from responding to the foils. The scene remained on the screen for a further 1000 msec after object presentation, providing a response window.

No memory was required to perform the spatial orienting task, but memory for the spatial context of target items could be used to facilitate target detection when the location of the target object in the learning task matched its location in the orienting task. On half of the trials, the object (32 target objects, 4 foils) appeared at the original learnt location. Memory for the object location from the scene cues therefore provided valid spatial information. In the other half (32 target-objects, 4 foils), the object appeared at an unlearnt, invalidly cued location (invalid), in the opposite hemifield. Scenes and object locations were counterbalanced across participants, so that objects were equally likely to occur in the left and right side, and in valid and invalid cueing conditions.

The orienting task amounted to a simple speeded target-detection task. To explore memory-guided visual attention in this context, we analysed RTs. The false-alarm rate was also calculated using foil trials.

#### 3.2.3 Explicit retrieval

**Fig. 3:**
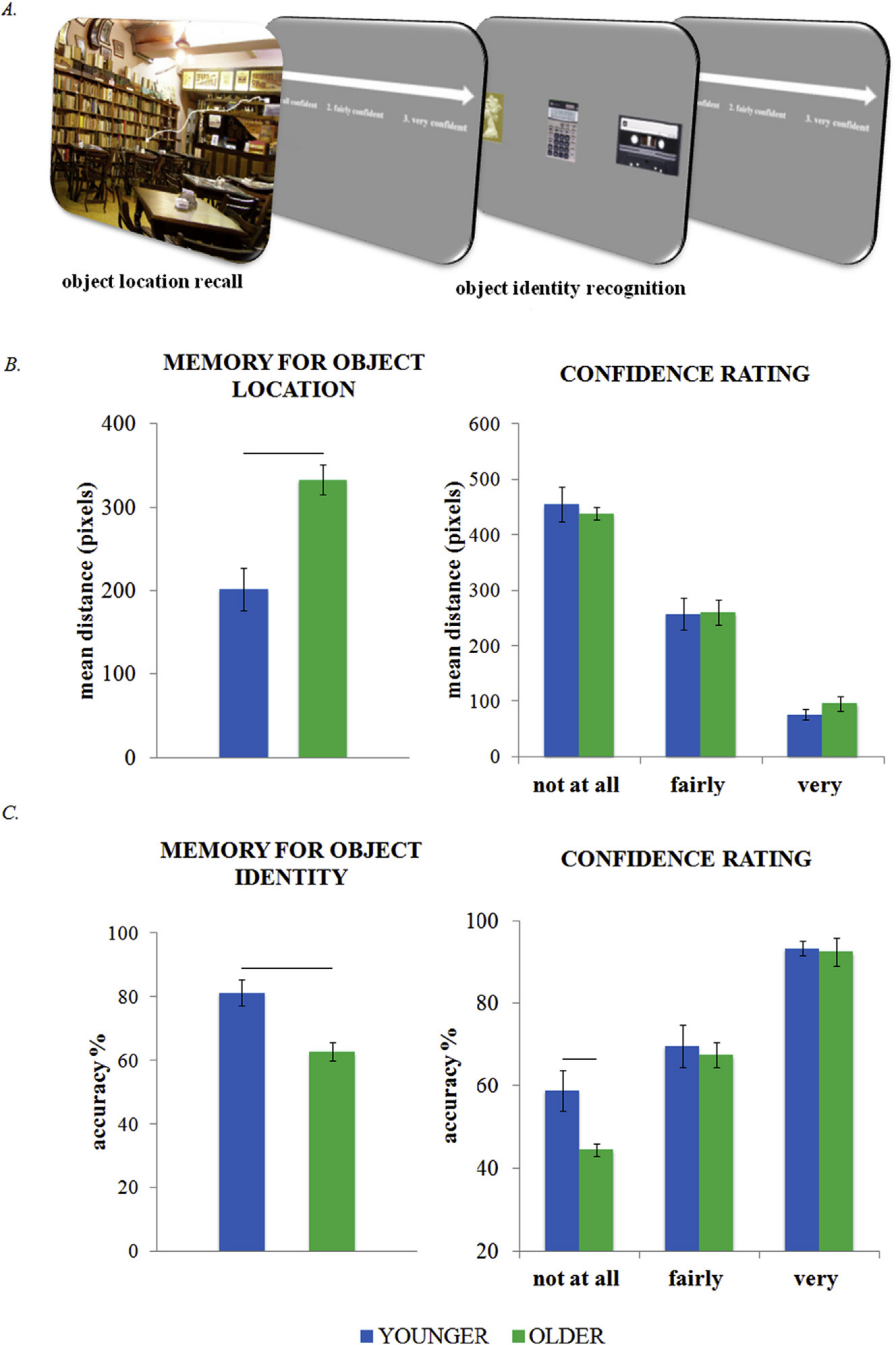
Memory session: experimental paradigm and results. (A) Schematic illustration of the explicit retrieval task. Participants placed the mouse cursor on the spatial location of the object associated with a specific scene, and estimated the confidence for the spatial memory performance. Then they chose the object associated with that scene and rated their level of confidence. (B) Results of memory for object location. Younger participants were more precise in the spatial memory task than the older participants. The awareness for the memory performance increased as the reported spatial location was closer to the veridical one. (C) Results of memory for object identity. Younger participants were more accurate than older in reporting the correct object associated with its scene in the 3AFC task. Participants were more accurate as a function of their awareness for the memory performance. In the case of lowest confidence ratings, older participants were less accurate than younger participants.

**Fig. 4:**
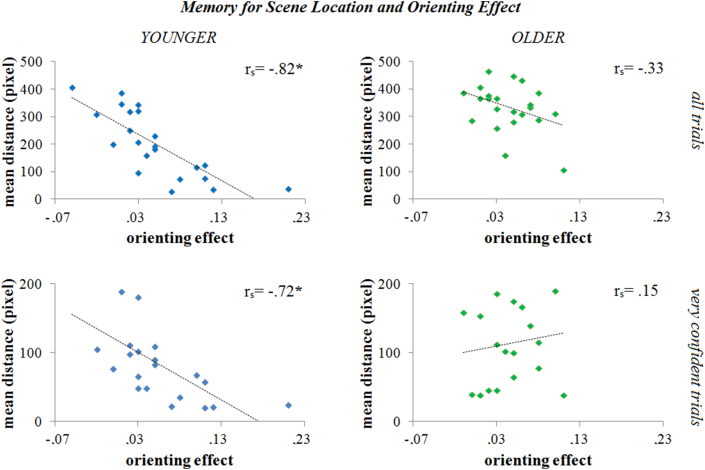
Relationship between explicit memory for object location in scene and the orienting effect. The graphs show the correlation between the magnitude of the orienting effect and the mean distance from the actual object location in all trials (upper panels) and for the “very confident” trials only (lower panels). A significant correlation was found in the younger group (left panels) but not in the older (right panels).

Immediately after the orienting task, participants were tested on their explicit memory for the location and identity of the object associated with each scene in the learning session. They viewed the 96 studied scenes in randomised order. In each trial, a scene appeared without the object embedded in it (Fig. 3A). Participants were instructed to place the mouse cursor in the remembered object location, within a 2-min time window. Following their response, they rated their confidence level in locating the object using the left (1), middle (2), or right (3) mouse button to indicate 1 = “not at all confident”, 2 = “fairly confident”, and 3 = “very confident”. A blank fixation period (1000–1500 msec) followed this rating. Three objects then appeared on the screen aligned horizontally (each within a transparent box of 150 × 150 pixels). Participants chose the object they remembered to be associated with the scene in this three alternative forced-choice recognition (3AFC) task. They used the left, middle, or right mouse button indicating respectively the object at left, middle or right position on the screen. For all trials, the competing objects were previously associated with other scenes. Competing items were drawn randomly from the pool of all objects. Each object appeared as the target associated with the scene only once but as a competing item on two other, randomly assigned scenes. The participants again rated their confidence level in their memory for object identity (Fig. 4A). No feedback was provided in this phase.

In this session, 72 out of 96 scenes had also been used in the previous orienting task. The remaining set of 24 learned scenes that were not presented in the orienting session enabled us to measure the quality of explicit retrieval of associations formed in the learning task in a way that was uncontaminated by any re-exposure to objects in previously learnt or unlearnt locations during the orienting task.

As a measure of explicit contextual memory for object location within a scene, we analysed the mean distance between retrieved location and actual object location (in pixels). As a measure of rough spatial memory for the hemifield of the object location, the mean accuracy for the side of the object was also calculated. The mean accuracy in the 3AFC task provided a measure of explicit contextual memory for object identity. Confidence ratings were analysed to assess awareness for the quality of explicit memory.

#### 3.2.4 Apparatus

The tasks were programmed using Presentation (Neurobehavioural Systems, Albany, NY). A personal computer controlled the stimulus displays and collected the responses. The stimuli were displayed on a 24-inch monitor with a resolution of 1028 by 768 pixels and a 60-Hz refresh rate.

## 4 Results

### 4.1 Learning object–scene associations

The level of accuracy in locating the objects was high in both groups. Both groups showed greater then 97% accuracy on average from the first block. A mixed ANOVA was performed with Search Accuracy as the dependent variable, Age (younger, older) as a between-subjects factor, and Blocks (1, 2, 3, 4) as a within-subject factor. Overall, the pattern of results showed very high levels of performance for both group, though also indicated that older participants took longer to reach the near-perfect accuracy of the younger group. Age and the linear contrast of Blocks interacted significantly [*F*(1,42) = 8.2, *p* = .007], suggesting steeper improvements in accuracy in the older participants. We followed up this interaction with a repeated-measure ANOVA with Block (1, 2, 3, 4) as within-subjects factor in each group. Results showed a significant linear effect of Block in the older group [*F*(1,42) = 14, *p* = .001] but not in the younger group [*F*(1,42) = 1.1, *p* = .323], indicating that older participants had a significant improvement of accuracy over the blocks, whereas younger participants were close to ceiling across all learning blocks (Fig. 1B). Additional Bonferroni-corrected post-hoc *t*-tests comparing Search Accuracy on different blocks between groups showed a difference on the first block [*t*(42) = 4.352, *p* = .002], in which younger participants (*M* = .995, *SE* = .01) were more accurate than the older participants (*M* = .979, *SE* = .03). By the end of the training, however, performance was equivalent and nearly 100% for both younger (*M* = .997) and older (*M* = .994) participants. In addition to the interaction, main effects of Age [*F*(1,42) = 14.0, *p* = .001] and Block [*F*(3,42) = 5.9, *p* = .001, linear contrast: *F*(1,42) = 14.3, *p* < .001] were also observed.

A mixed ANOVA compared Search Times between Age groups (younger, older) across different Blocks (1, 2, 3, 4). Trials in which the object was not found during the learning task were excluded from this analysis (younger: .4%; older: 1.2%). The results indicated that search times decreased progressively and similarly for both groups, though older participants were slower overall (Fig. 1C). There was no interaction between Age and Blocks [*F*(3,42) = .3, *p* = .800]. Search times decreased linearly for both groups [main effect of Block: *F*(3,42) = 18.3, *p* < .001, linear contrast of Block: *F*(1,42) = 26.8, *p* < .001]. Older participants had significantly slower search times [main effect of Age: *F*(1,42) = 14.2, *p* = .001]. Mean Search Times for older participants were 2482 msec (*SE* = 139 msec) in Block 1 and 1429 msec (*SE* = 270 msec) in Block 4; Mean Search Times for the younger group were 1910 msec (SE = 172 msec) in Block 1 and 826 msec (*SE* = 80 msec) by Block 4. As an additional, and possibly more sensitive measure of changes in search times, we also compared their slopes using regression functions. Search time slopes also showed no difference between groups in an independent *t*-test [*t*(42) = −.2, *p* = .811].

### 4.2 Memory-based orienting

Our primary measure of memory-guided attention was RTs to detect the appearance of the target object, presented at either a learned (valid) or unlearned (invalid) location. The scenes for which participants failed to find the object in the third and fourth learning blocks were excluded from the analysis. RTs below 200 msec or above two standard deviations from the mean were also excluded from analysis. The average percentage of trials discarded for both younger and older participants was 4%. A mixed ANOVA was performed with Age (younger, older) as a between-subjects factor and Validity of the memory target location (valid, invalid) as a within-subject factor. Analysis of RTs during the Orienting task showed significant and similar benefits of memory-guided orienting in both age groups (Fig. 2B). There was no interaction between Age and Validity [*F*(1,42) = .11, *p* = .747]. Both main effects were significant. Participants responded significantly more quickly to objects that appeared in valid (*M* = 411, *SE* = 12 msec) than in invalid (*M* = 453, *SE* = 14 msec) locations [main effect of Validity: *F*(1,42) = 47.1, *p* < .001]; and the older group had slower RTs (*M* = 476, *SE* = 18 mecs) than the younger group (*M* = 388, *SE* = 18 msec) overall [main effect of Age: *F*(1,42) = 12.1, *p* = .001]. To ensure that the different response speeds between the two groups did not mask any qualitative difference in orienting effects, we also calculated normalized measures of the validity effect [(invalid − valid)/(invalid + valid)]. Normalized orienting effects were equivalent between groups [one-way ANOVA (*F*(1,42) = .19, *p* = .663)].

We used a Bayesian analysis to test whether there was greater evidence for older and younger participants showing equivalent or different degrees of memory-based orienting effects. The Bayesian method assumes that the null hypothesis (H0) and the alternative hypothesis (H1) are equally plausible a priori, and provides graded evidence for each hypothesis in turn (Wagenmakers, 2007). To compute the Bayesian Information Criteria (BIC), we used the simple formula from Masson (2011) which requires as inputs: the number of independent observations, the sum of squares for the effect of interest, and the sum of squares for the error term associated with it. This method enables inferences about whether the evidence favours the H0 or the H1, with values ranging from 0 (i.e., no evidence) to 1 (i.e., very strong evidence) (for further details see also Patai, Buckley, & Nobre, 2013). The Bayesian analysis strongly supported the null results we obtained as providing a better account of the results (*H0* = .87; *H1* = .13).

Given the simplicity of the task, and the potential concern that participants might be inclined to respond without perceiving or discriminating the appearance of the object, we also analysed false alarms. There was no significant difference in the rates of false alarms [*F*(1,42) = .03; *p* = .860] between younger (*M* = .18, *SE* = .04) and older (*M* = .19, *SE* = .05) participants, indicating that both groups were able to inhibit their response to the foil stimulus.

### 4.3 Explicit retrieval

In order to test whether previously reported impairments in explicit contextual memories with ageing were present in our sample, we compared explicit contextual memory for object locations and object identities after the orienting task in the two groups of participants.

One potential concern, however, was that the re-occurrence of target objects in their studied (valid) or novel (invalid) locations within the orienting task could contaminate the findings. For this reason, we compared the spatial-location and object-identity memories in the 24 ‘pure memory’ scenes with memory in the scenes appearing in the valid and in the invalid conditions in the orienting task in the two groups of participants. A mixed-effects ANOVA showed no main effect of scene type (pure, valid, invalid) [spatial location: *F*(2,42) = .36, *p* = .700; object identity: *F*(2,42) = .66, *p* = .517)] or interaction between scene type and age group [spatial location: *F*(2,42) = 1.4, *p* = .240; object identity: *F*(2,42) = 1.4, *p* = .248)]. A main effect of age group for both spatial location [*F*(1,42) = 18.8, *p* < .001] and object identity [*F*(1,42) = 12.1, *p* = .001] memory confirmed the deficit in both types of explicit recall. Additionally we compared explicit memory using only pure memory trials. Results showed that younger participants (*M* = 191.3, *SE* = 24) were more accurate in indicating the location of objects on the scenes compared to older (*M* = 331.8, *SE* = 24), [*F*(1,42) = 17.1, *p* < .001]. We found the same pattern in the case of memory for object identity, for which older participants (*M* = .66, *SE* = .04) were less accurate than younger (*M* = .80, *SE* = .04) [*F*(1,42) = 1.4, *p* = .008]. In light of these results, performance measures based on all 96 scenes were used in subsequent analyses.

#### 4.3.1 Explicit memory for object location

In order to explore the quality of the spatial memory available for guiding the orienting effect, we measured the distance (in pixels) between the position reported by the participants in the explicit retrieval task and the veridical object location in the learning phase. This measure provides an estimate of spatial-memory precision.

The group comparison using a one-way ANOVA indicated better performance in the younger (*M* = .81, *SE* = .02) than in the older group (*M* = .63, *SE* = .04) [*F*(1,42) = 14.3, *p* < .001]. Comparing mean-pixel distances between the retrieved and actual studied object location showed that younger participants were reliably closer (*M* = 201.5, *SE* = 25) than the older participants (*M* = 333.3, *SE* = 18) [*F*(1,42) = 18.3, *p* < .001] (Fig. 3B).

To follow up on the group differences in the spatial memory task, we examined the frequency of confidence ratings. A non-parametric two-samples Kolmogorov–Smirnov test was performed on the number of responses across the three confidence ratings for spatial and object-based memory measures. Mirroring their explicit memory performance, younger participants chose the “very confident” rating more frequently than the older participants (*z* = 1.9, *p* = .001), while older participants chose the “not at all confident” rating (*z =* 2 = .5, *p* < .001) more frequently. No difference was found on the “fairly confident” rating (*z* = 1.2, *p* = .109).

Additionally, we analysed the mean distance as a function of confidence rating in the two groups in order to compare alignment between the objective, actual spatial memory accuracy and the subjective experience of remembering. In this case, because the number of responses in each confidence-rating band could vary, we also performed *F*-test permutation to follow-up any significant effect. Standard and permutation *p* values are both reported (Ernst, 2004, Maris et al., 2007, Rohenkohl et al., 2014). As a first step, we calculated the mean difference between conditions of interest using an ANOVA. We subsequently tested for significance of the resulting *F*-value by comparing it to a null distribution generated using a Monte-Carlo simulation. This null distribution was obtained by randomly shuffling the condition labels within each participant's data before calculating the F value, and then repeating this process 10,000 times. The *F*-test permutation *p* value was determined as the proportion of random partitions that resulted in a larger test statistic than the observed one.

An ANOVA compared mean distance between Age groups (younger, older) as a function of Confidence Rating (very confident, fairly confident, not at all confident). We found that distance varied as a function of confidence rating [main effect: *F*(3,42) = 129.6; *p* < .001; confirmed by *F-test* permutation, *p* < .001] Predictably participants were closer to the actual target location when they were more confident. No main effect of Age or interaction between Age and Confidence Rating was found (Fig. 3B). These results showed a strong congruence between confidence rating and quality of spatial memory in both groups, suggesting no deficit in memory awareness, or meta-memory, in older participants.

Because the invalid trials in the orienting task always appeared on the opposite hemifield, orienting benefits may have relied on the sparing of a coarse spatial memory for object side. We therefore also compared memory accuracy for the side (left *vs* right) of the object, and confirmed a significant deficit in older (*M* = .68, *SE* = .02) relative to younger (*M* = .82, *SE* = .02) participants [*F*(1,42) = 16.2; *p* < .001] even when these coarse measures were taken.

#### 4.3.2 Explicit memory for object identity

To compare explicit contextual memory for object identity between groups we used accuracy in a three-alternative forced-choice task. The one-way ANOVA indicated that the younger group (*M* = .81, *SE* = .04) was significantly more accurate than the older group (*M* = .63, *SE* = .02) [*F*(1,42) = 14.3; *p* < .001] (Fig. 3C). Confidence ratings followed explicit memory measures for object memory. Younger participants chose the “very confident” rating more frequently (*z* = 1.8, *p* = .003), while older participants were more prone to choosing the “not at all confident” rating (*z =* 2 = .4, *p* < .001). No difference was found on the “fairly confident” rating (*z* = .9, *p* = .387).

An ANOVA comparing accuracy between Age groups (younger, older) as a function of Confidence Rating (very confident, fairly confident, not all confident) showed a significant linear effect of Confidence Rating [*F*(1,42) = 195, *p* < .001; confirmed by *F-test* permutation, *p* < .001] participants were more accurate as a function of their increased confidence. There was no main effect of Age [*F*(1,42) = 1.9, *p* = .177], but the interaction between Age and Confidence Rating was significant [*F*(3,42) = 6.3, *p* = .016; confirmed by *F-test* permutation, *p* = .02]. Post-hoc analysis indicated that when participants were “not at all confident” about their memory performance, older participants (*M* = .45) were less accurate than younger (*M* = .59) (Fig. 3C). The pattern of results suggests a spared awareness for the quality of the explicit memory in older participants also in the case of memory for object identity.

#### 4.3.3 Relationship between explicit contextual memory and orienting effect

In order to probe the extent to which explicit spatial and object-based contextual long-term memory predict the benefits of attention in the orienting task, we correlated the magnitude of the normalized orienting effect with the memory scores separately in each group. As before, the orienting effect was calculated on 64 scenes, excluding foils trials. For measures of explicit memory, we used the same 64 scenes from which orienting effects were derived. We calculated the mean distance from the actual object location (memory for scene location) and the accuracy of the 3AFC task (memory for scene object) based on the explicit retrieval performance achieved on these same 64 scenes. The set of 64 scenes contained an equal number of valid and invalid targets in the orienting task. The pattern of results was equivalent if all 96 scenes were used to derive explicit memory measures. Correlation analyses were based on non-parametric Spearman's rho, which circumvents any possible issues with outliers. The Fisher *r*-to-*z* transformation was used to compare correlation coefficients between the younger and older groups (Cohen, Cohen, West, & Aiken, 2013).

First, we looked at correlations between spatial memory and orienting. In the younger group, we observed a significant and strong negative correlation between the magnitude of the normalized orienting effect and the mean distance from actual object location in the explicit spatial memory task [*r*_*s*_(20) = −.82; *p* < .001]. In other words, more precise explicit spatial memory (smaller distance) resulted in a larger orienting effect. No such correlation occurred in the older group [*r*_*s*_(20) = −.33; *p* = .136] (Fig. 4). The correlation coefficients differed significantly between groups (*z* = 2.5; *p* = .01).

We worried about whether the lack of correlation between explicit memory and orienting in the older participants might be driven by the overall worse memory in this group. In order to check for a possible influence of impaired memory performance on the relationship between explicit memory and orienting effect, we repeated the analysis including only “very confident” rating trials. We applied a permutation test shuffling the group label via a Monte Carlo simulation (10,000 repetitions), in order to deal with smaller trial numbers on the highest confidence rating for older compared to younger participants. Results in this follow-up analysis still showed a significant negative correlation between the orienting effect and the spatial memory in the younger [*r*_*s*_(19) = −.72; permutation test: *p* < .001; one participant never rated highest confidence] but not in the older group [*r*_*s*_(18) = .15; permutation test: *p* = .633; two participants never rated highest confidence] (Fig. 4). Correlation coefficients between the younger and older groups were significantly different (*z* = −3.1; *p* = .002).

**Fig. 5:**
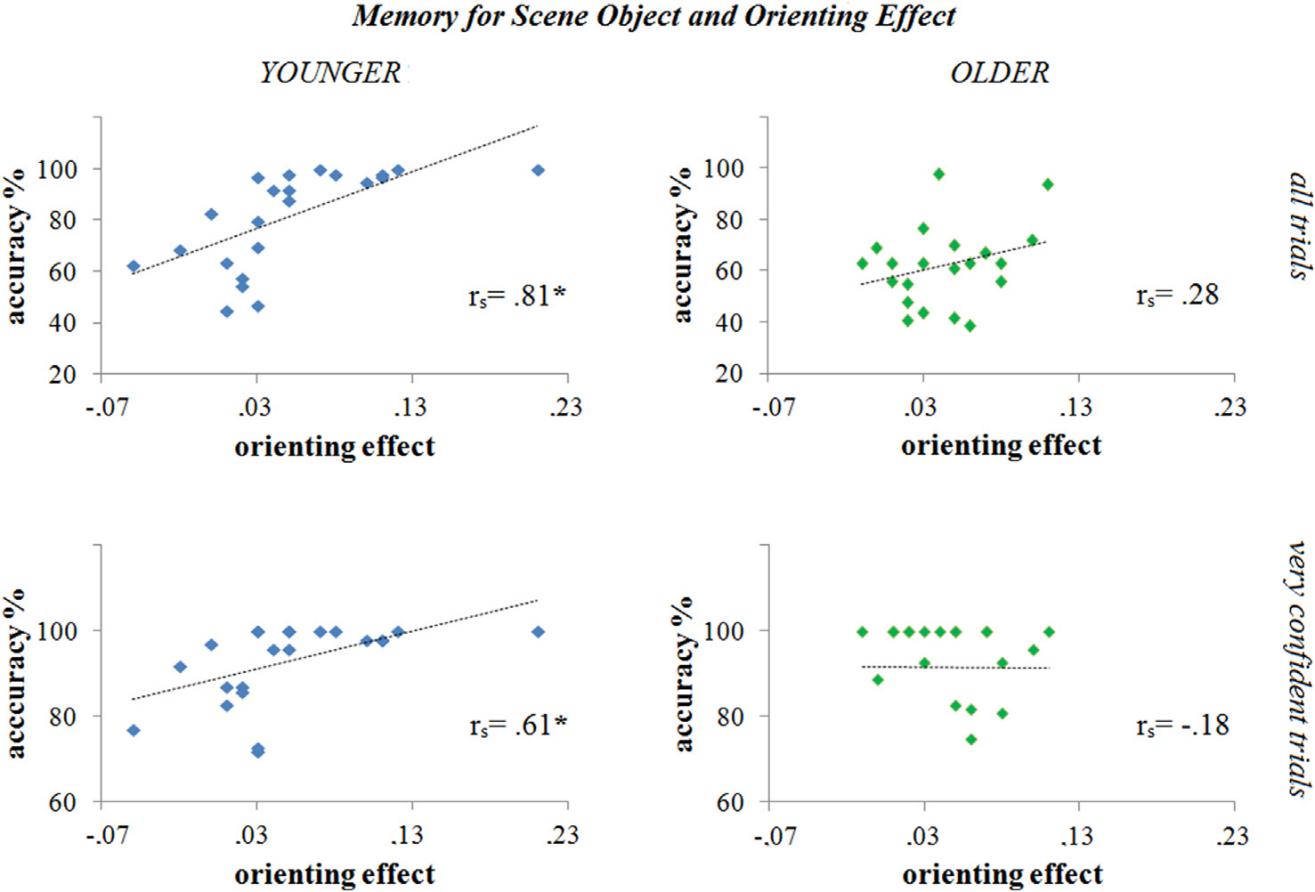
Relationship between explicit memory for the object associated with the scene and the orienting effect. The graphs show the correlation between the magnitude of the orienting effect and the accuracy for the memory of what object was associated with the scene on all trials (upper panels) and on the “very confident” trials only (lower panels). In the younger group (left panels), there was a strong correlation between orienting effect and memory for the scene object. No correlation was found for the older group (right panels).

A similar pattern was observed when we correlated the magnitude of the orienting effect with the accuracy of the memory for the object associated with the scene. The two measures showed a strong positive correlation in the younger group [*r*_*s*_(20) = .81; *p* < .000] but not in older group [*r*_*s*_(20) = .28; *p* = .206]. The correlation coefficients differed significantly between groups (*z* = 2.6; *p* = .01). Analyses of correlations using only “very confident” trials showed a similar pattern. Memory for object identity was positively correlated with the orienting effect in the younger group [*r*_*s*_(20) = .61; permutation test: *p* < .001], but not in the older group, for which we found a non-significant negative correlation [*r*_*s*_(19) = −.18; permutation test: *p* = .204; one participant never rated highest confidence] (Fig. 5). Again, there was a difference between groups on the correlation coefficients (*z* = 2.7; *p* = .006).

## 5 Discussion

Using a novel experimental task, we replicated the well-established deficit of explicit recall for the location and identities of objects associated with scene contexts with ageing (Chalfonte and Johnson, 1996, Kessels et al., 2007, Kessels et al., 2010, Meulenbroek et al., 2010, Old and Naveh-Benjamin, 2008). Surprisingly, however, we showed that the ability to use these contextual memories to guide attention remained intact. Also intact were measures of individuals' knowledge about the quality of their explicit memories.

In line with previous demonstrations of explicit contextual memory impairments in older people (Bender et al., 2010, Cooney and Arbuckle, 1997, Denney et al., 1992, Meulenbroek et al., 2010, Naveh-Benjamin et al., 2003, Old and Naveh-Benjamin, 2008, Spencer and Raz, 1995) we found that older adults were impaired in recalling contextual memories in our task. Compared to the younger group, they showed poorer explicit memory for both object locations and object identities. We also showed that older adults were able to monitor their explicit forgetting. Indeed, the self-rated confidence was a valid reflection on their performance, as in the younger group. It has been demonstrated that meta-memory is preserved in elderly individuals, despite lower levels of explicit memory performance on the same learnt material (Halamish, McGillivray, & Castel, 2011), though this outcome has been suggested to depend on high education levels (see Szajer & Murphy, 2013).

The deficits displayed by older participants in recalling locations and object identities associated with scenes were unlikely to reflect strong deficits during the learning task. Both older and younger participants displayed excellent performance during the learning task, with accuracy about 97% even within the first learning block. Performance of younger participants was significantly better in the first learning block, although both groups had near-perfect performance by the end of the learning session. In addition, even though the older group had slower search times, their search times improved consistently, and at an equivalent rate as that of the younger cohort, through learning blocks.

Nevertheless, it is not possible to ensure the encoding process was unaffected by ageing. Although older participants reached near perfect accuracy in the learning task, their encoding process might, for example, have been adversely affected by other information present in the natural scenes, irrelevant to the goal of our task, which older adults may have found more difficult to ignore (Biss et al., 2013, Campbell et al., 2012, Healey et al., 2008). Subtle deficits in encoding, therefore, may have contributed to the deficits in explicit memory retrieval observed in older adults.

Interestingly, and counter to our initial prediction, the significant impairments in explicit memory for object location and identity within scenes were not accompanied by any deficit in memory-based orienting of attention. Older participants showed reliable orienting effects that were equivalent to those in younger participants. In both groups, items appearing in previously learnt locations, for which the scenes provided valid memory cues, were detected with faster RTs than items appearing at other, unlearnt locations, for which the scenes provided invalid memory cues. The magnitude of the normalized orienting effect was the same between groups, confirming the robustness of the memory-based orienting mechanism in normal ageing.

The discrepancy between impaired explicit retrieval and spared orienting in the older group could not be explained by a difference in the specificity with which spatial memories were maintained. Arguably, a coarser spatial memory that merely preserved object side might have been sufficient to support an orienting benefit based on spatial memories. However, even the coarsest measure of explicit spatial memory for object side was significantly impaired in the older group.

Notably, our correlation analyses showed that whereas in younger participants explicit recall for object location and object identity within scenes strongly predicts performance benefits from memory-based orienting, no such correlations are observed for older participants. There are multiple possible explanations for this dissociation.

One possible explanation is that explicit contextual memories are not necessary to guide memory-based perceptual enhancements. Instead, there may be other, implicit forms of contextual memories that are preserved, which rely on different memory systems, that can guide perceptual functions (Chun, 2000, Chun and Jiang, 1998, Hutchinson and Turk-Browne, 2012). Evidence for dissociation between a spared implicit and deteriorated explicit memory in ageing has been well documented (Chiarello and Hoyer, 1988, Dennis and Cabeza, 2008, Gopie et al., 2011, Howard and Howard, 2013, Kessels et al., 2005, Nemeth et al., 2013, Russo and Parkin, 1993, Ward et al., 2013).

An alternative possibility is that the engrams of the memories that guide explicit recall of contextual memories and that guide orienting of attention may be the same, and rely on the same memory systems, but the ability to use this mnemonic information for different purposes may be differentially impaired in ageing (Cohn, Emrich, & Moscovitch, 2008). Whereas the ability to access these engrams for explicit, conscious retrieval mechanisms may become faulty with ageing, the ability to use these memories implicitly to improve performance on target detection tasks may be preserved (Howard et al., 2005, Merrill et al., 2013, Neider and Kramer, 2011, Schmitter-Edgecombe and Nissley, 2002).

Differences in strategies used by the two age groups in different phases of the experiment may also have lead to different degrees of association between the memory-based orienting of attention and explicit memory. For example, cognitive differences in the encoding style may have affected subsequent retrieval (Friedman et al., 2007, Naveh-Benjamin et al., 2007). Older participants may have memorized the object identity and its spatial location on a particular natural scene relying on different strategies (e.g., semantic or visual) (Craik and Lockhart, 1972, Craik and Simon, 1980, Kuo et al., 2015). We did not collect any qualitative data on the encoding or retrieval approaches used by our participants, and therefore cannot rule out strategy differences.

Finally, it is also worth noting that we found overall slower performance of the older group compared to the younger group in all phases of the experiment. This decreased perceptual speed of processing has been amply documented in previous studies, and is considered a typical consequence of ageing (Albinet et al., 2012, Salthouse, 1996, Salthouse, 2000, Salthouse, 2011). Interestingly, however, the response slowing in the current study did not interact with measures of learning or memory-based orienting.

At this point, the precise nature of the memory (or memories) guiding attention in older participants remains to be investigated. The role of explicit and implicit contextual memory in orienting visuo-spatial attention will require further individuation. In order to weigh the contribution of explicit memory in orienting visuo-spatial attention, it would be helpful to use the task described here, to assess patients affected by hippocampal damage with impaired explicit memory, or amnesic patients with middle temporal lobe damage. Further studies are needed to shed light on the neural system(s) involved, and on the contribution of medial temporal lobe structures to the proactive modulation of perception by contextual, associative memory in healthy ageing.
